# Effect of subconscious changes in bodily response on thought shifting in people with accurate interoception

**DOI:** 10.1038/s41598-023-43861-w

**Published:** 2023-10-03

**Authors:** Mai Sakuragi, Kazushi Shinagawa, Yuri Terasawa, Satoshi Umeda

**Affiliations:** 1 Department of Psychology, Graduate School of Human Relations, Keio University, 2-15-45 Mita, Minato-ku, Tokyo, 108-8345 Japan; 2 Keio University Global Research Institute, 2-15-45 Mita, Minato-ku, Tokyo, 108-8345 Japan; 3 Department of Psychology, Faculty of Letters, Keio University, 2-15-45 Mita, Minato-ku, Tokyo, 108-8345 Japan

**Keywords:** Psychology, Human behaviour

## Abstract

Our thought states shift from one state to another from moment to moment. The relationship between the thought shifting and bodily responses is yet to be directly examined. This exploratory study examined the influence of cardiovascular reactivity and interoception—sensing an internal bodily state—on the shifting of thought states. Participants (*N* = 100, 70 women) completed two tasks: the heartbeat counting task (HCT) and the vigilance task (VT). We assessed their interoceptive accuracy through their performance on the HCT. The VT was a simple sustained attention task in which participants pressed a key when the target stimulus appeared and were asked to report their thoughts. We presented subliminal vibration stimuli to induce alterations in heart rate (i.e., vibration block). Results showed that participants with higher interoceptive accuracy reported more continuation of self-referential thought (about past episodes and future plans regarding themselves) during the vibration block than did those with lower interoceptive accuracy. These results suggest that individuals with higher interoceptive accuracy are more likely to be influenced by their subliminal bodily response, resulting in divergent attention from the task and intermittent self-referential thought.

## Introduction

In our daily lives, we experience various states of thought, such as when something comes to mind unintentionally (spontaneous thought), when the content of our thoughts shifts from one thought to another (mind-wandering; MW), or when we continue to think deeply about a certain matter (goal-directed thought;^1^). What triggers such thought shifting has primarily been pursued in MW research. MW refers to the phenomenon of one’s thoughts wandering to things unrelated to the present context^2^. It has been estimated to occupy between 20 and 50% of our daily lives and mainly involves involuntary autobiographical memories^3^, spontaneous future thinking^4^, prospective memories^5^, and intrusive memories^6^. Various factors have been reported to contribute to the occurrence of MW. The difficulty and proficiency of action^7,8^, personal interest in tasks^8^, differences in individual working memory capacity (attentional resources needed to temporarily hold the information necessary to act)^9^, fatigue, boredom, and mood state during a task^10,11^ are factors that could contribute to the occurrence of MW. Moreover, the perception of external stimuli has received much attention as a trigger for thought shifting before and after MW. For example, most MW during simple cognitive tasks is triggered by irrelevant cue phrases presented on a monitor^3,12–15^. In these studies, task-irrelevant cue phrases representing positive, neutral, or negative things or actions were presented during the task while participants engaged in a simple attention task. Among MW contents reported during the task, involuntary memory recall was more likely to be triggered by negative phrases^3^. This suggests that negative cue phrases could serve some adaptive function by acting as a warning sign of what has happened before and protecting the individual from potentially harmful or unpleasant events. Contrastingly, cue stimuli strongly related to the task they were originally intended to address sometimes promote a return from MW. For example, during a lecture, students often recover from MW when they notice peripheral clues related to the class^16^. However, a major challenge in studying MW triggers is that MW often occurs unconsciously^17^. Indeed, more than half of all MW in daily life is prompted by unknown triggers^18^, suggesting that treating only consciously perceived external stimuli as triggers of thought transitions may be insufficient.

Recently, the impact of interoception—the sensing of the internal bodily state—on thought shifting has also been studied. Interoception is the overarching mechanism by which the nervous system detects and integrates signals originating from within the body, primarily from internal organs, and functions at both conscious and unconscious levels^19,20^. Most of our interoception, such as monitoring our own psychophysiological state, unfolds at an unconscious level and often only reaches consciousness when there is a deviation in the system (i.e., pain, thirst)^21^. However, subconsciously processed interoceptive signals amplify or attenuate specific perceptual and cognitive processes^22^. For example, the detection rate of visual, tactile, and facial expression stimuli varies depending on the phase (systole/diastole) of the cardiac cycle during which the stimuli are presented^21,23,24^. It is also associated with physiological functions, such as ingestion and elimination^25^, as well as cognitive functions such as memory, emotion^26^, decision-making^27^, and time perception^28^. In addition, individuals with more accurate cardiac interoception; that is, more accurate detection of cardiac activity, may be better able to detect stimulus-induced autonomic activity and apply it to subsequent adaptive cognition and behavior^29^. For example, interoceptive accuracy is associated with sensitivity to specific emotional expressions of others^30^ and the magnitude of the effect of reappraisal on the control of disgust^31^. Moreover, individuals with more accurate interoception are less susceptible to the adverse effects of the cardiac cycle on memory^32^. Also, they can better recall prospective memory at the appropriate timing in response to cue stimuli^33^.

Several studies have shown a correlation between MW and interoception. First, brain regions activated during MW include the posterior insular cortex, in addition to the default mode network associated with resting state, and the fronto-parietal network related to executive function^34^. As this region is associated with sensing interoceptive information^35,36^, interoception could be related to the specific contents of MW, such as body representations. The self-relevance of thoughts during MW is also known to covary with the information processing of heartbeat signals in the default mode network and right insular cortex^37,38^. Both regions monitor body signals and autonomic regulation, suggesting that neural responses to body signals may encode specific contents in MW. Furthermore, interoception might also play a role in awareness of thought shifting. For example, the salience network, which includes insula, is activated during awareness of MW during respiratory focus tasks^39,40^. The salience network is involved in autonomic processing and in detecting the saliency of external/internal events^41–43^, and these functions could be important for noticing changes in one’s own thoughts. However, whether changes in interoception cause thought transitions in the same way as exteroception, or what individual differences exist in the influence of interoception on thought transitions, remains unknown.

To directly examine the relationship between interoception and thought shifting in a controlled experimental environment, we considered it necessary to manipulate participants’ bodily responses and interoception through external manipulation. The presentation of supraliminal visual, auditory, and vibration stimuli to body parts can alter participants’ heart rate^44–47^; their interoception as the ability to detect heart rate^48,49^; or cognitive functions related to interoception such as time perception^50^, emotion evaluation^51,52^, and decision-making^53^. However, in these studies, participants could consciously perceive the stimuli for manipulating heart rate, or received explicit feedback about their own interoception. In experiments using such threshold stimuli or explicit instructions about bodily sensations, it is impossible to isolate the effect of the change in internal bodily state on thought shifting since awareness of the stimuli could cause the thought state to shift in the first place. Therefore, we attempted to determine whether subliminal tactile stimuli could induce changes in heart rate at an unconscious level during the task. Subliminal tactile stimuli can modulate the detection rate immediately following suprathreshold tactile stimuli^54,55^ or facilitate the perception of subsequent visual stimuli or motor responses^56,57^. Such effects have been applied to techniques for optimizing driver attention by presenting subthreshold or subthreshold-like minute vibration stimuli during driving^58–61^. In addition, the detection rate of subliminal tactile stimuli varies depending on the relationship between the cardiac or respiratory cycles and the timing of stimulus presentation^62,63^. However, it is unclear how subliminal tactile stimuli affect physiological responses such as cardiac activity and higher-order cognitive functions such as memory, emotion, and thought.

This exploratory study examined the possibility that unconscious interoceptive information processing could cause changes in thought, which has yet to be clarified in previous studies. We also examined the influence of the ability to detect fluctuations in cardiovascular reactivity occurring in real time on the transition of thought states. Therefore, among the subcategories of interoception, we focused on interoceptive accuracy, which is objective exactness in detecting internal bodily sensations^64^. To measure this indicator, participants completed the heartbeat counting task (HCT)^65^. Performance on this task correlates with the strength of functional coupling in areas related to interoception processing at rest^66^ and the amplitude of afferent signals associated with cardiac activity^67^. This suggests that the interoceptive accuracy measured by the HCT is also reflected in the processing of heart rate changes when attention is not focused on bodily responses. Participants also completed the vigilance task (VT). The VT is a simple attention task and is one of several used to measure spontaneous shifting of thought states in the laboratory^2^. Participants reported their thought contents, contemplation (how deeply they thought), and consistency (coherence of thought contents) while performing a task in which they responded by pressing the keyboard only when the target stimulus was presented. The thought probe we used to record participants’ thought contents included six categories based on the semantic relevance of the thought contents to the task or the stimuli presented during the task^68^. Based on our daily experience of thought states, transition, in which the content of reported thought changes between trials, and continuation, in which the same content is reported between trials, were the subjects of the investigation. Additionally, during the VT, we presented subliminal vibration stimuli to induce implicit changes in participants’ heart rates. The reasons we used subliminal vibration stimuli were (1) they are suitable for subliminal presentation because they are less invasive, and (2) supraliminal slight vibration stimuli can change the heartbeat itself and emotional responses^44–46^. We examined how thought shifting (i.e., transition and continuation) varies depending on the interoceptive accuracy of the individual, as measured by the performance of the HCT, and the presence or absence of heart rate change induction by vibration presentation. The hypotheses were that (1) changes in heart rate by vibration would induce thought shifting, and (2) the effect of (1) would depend on one’s interoceptive accuracy.

## Material and methods

### Participants

One hundred university and graduate students (70 women, 30 men, *M*_*age*_ = 21 ± 1.46 years) were recruited. All participants had normal or corrected-to-normal vision. This study was approved by the Keio University Research Ethics Committee (no. 210300000) and was conducted per the Declaration of Helsinki. All participants provided written informed consent before participation. To examine the effect of subliminal vibration presentation on heart rate, we did not explain the presentation of vibration during VT in our pre-experiment briefing. After the experiment, we disclosed that we were presenting subliminal vibration during the VT and the purpose of it, and again acquired written informed consent for the use of the data obtained in the experiment.

### Apparatus

We used a pulse oximeter (NONIN, 8600) and a cuff (NONIN, 8000SM), attached to the tip of the left index finger, to measure participants’ pulse rates. The measured signal was recorded by POWER1401mk2, and Spike2 (Cambridge Electronic Design). Participants could not know their own real-time pulse rate by viewing the waveform of their own pulse or listening to sounds linked to their pulse rate. The heart rate index used in this study is the temporal variation in the interval between the apexes of participant's pulse wave at rest (see “Heart Rate Variability (HRV)”), which can be measured accurately enough with a pulse oximeter. Therefore, considering the burden of attaching a measurement device such as an electrocardiogram (ECG) to participants’ bodies, we used a pulse oximeter, which is relatively simple compared to an ECG.

**Figure 1:**
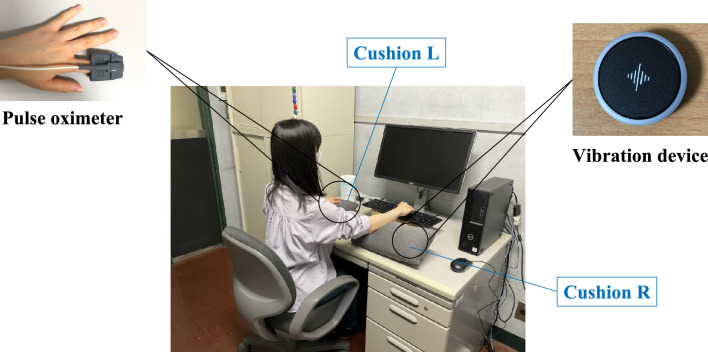
The arrangement of the apparatus during the experiment. Participants placed their left and right arms on Cushions L and R, respectively, and a pulse oximeter was attached to the left index finger. We set the vibration device in the right half of Cushion R.

A vibration device (Soundbrenner_Pulse_615867123689), connected via Bluetooth to an iPad, was used to present the subliminal vibration stimuli. The experimenter operated the device from behind a partition, out of sight of participants, using the Soundbrenner application on the iPad. The vibration stimuli presented during the experiment were the smallest of the three levels of vibration intensity and duration that could be set for the vibration device. Soundbrenner was designed for music performance, and the values for vibration intensity, frequency, and stimulus presentation time could not be set as exact values. We used two cushions as armrests (Cushion L and R). Both were made by Seria Co., Ltd., in Japan, whose surface cover was made of polyester, and the inner stuffing was made of polypropylene, measuring 17 cm in width and 37 cm in length. Participants placed their left forearm on Cushion L and their right elbow on the left half of Cushion R. To keep the device out of sight, it was inserted into the right half of Cushion R. The vibration stimuli were presented to the right elbow through a Cushion R. We placed Cushion L to secure the left hand, which was wearing a pulse oximeter and to prevent participants from being suspicious of Cushion R, which was used to hide the vibration device. The arrangement of the apparatus used during the experiment is shown in Fig. 1.

### Procedure

**Figure 2:**
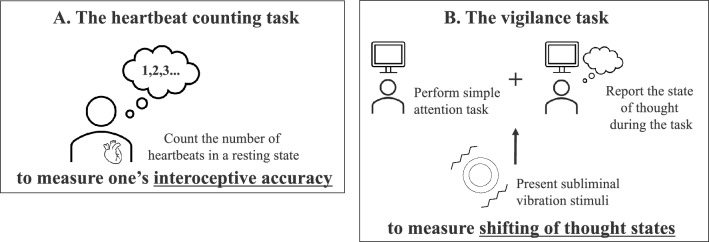
(**A**) and (**B**) Flow of the tasks in this experiment. Participants performed two tasks: the heartbeat counting task (**A**; the HCT) and the vigilance task (**B**; the VT). We used the former task to measure participants’ interoceptive accuracy and the latter to measure the shifting of thought states during the task.

Participants came to the laboratory and performed two tasks: the HCT and the VT. In both tasks, we measured participants’ pulse rates with a pulse oximeter (Fig. 2).

#### The HCT

Participants first completed the HCT^65^ to measure their interoceptive accuracy (Fig. 2A). This task comprised three processes: resting heart rate measurement, the HCT, and the time estimation task (TET). In both the HCT and the TET, all cues for the start and end of the task were provided by the experimenter, and all participant responses were given verbally.

First, participants put a pulse oximeter on their left index finger and measured their resting heart rate for three minutes. They placed their entire left arm on Cushion L on the desk at that time.

In the HCT, they silently counted their own heartbeats over a series of time intervals (2 × 25 s, 2 × 35 s, and 2 × 45 s) and then reported the number of heartbeats counted for each interval. At that time, we instructed participants as follows:

In this task, you are asked to count your heartbeats at various time intervals. You will be asked to count your heartbeat when I say “Start.” Then, until I say “Stop,” count your own heartbeat without saying it aloud. Please do not touch any part of your body, press your body hard against a backrest or desk, or hold your breath to count your actual heartbeat. Do not count heartbeats that you did not actually feel by assuming that they are probably beating based on the sensations you felt before and after the heartbeats, or by estimating the number of heartbeats that occurred based on your knowledge of it and your sense of time. Please answer only the number of heartbeats you actually felt. If you could not feel any heartbeats, please answer “0.”

This instruction conforms to the adapted version, which asks participants to avoid reliance on signals that are not interoceptive and to report only the perceived heartbeat^69^.

Lastly, they estimated the length of the time they felt had elapsed at a series of time intervals (2 × 23 s, 2 × 49 s, and 2 × 56 s) and then reported that length (TET). In the HCT and TET, each interval was measured randomly.

#### The VT

##### Task flows

After the HCT, the experimenter verified that the vibration device and iPad were properly connected via Bluetooth. The experimenter then placed Cushion R containing the vibration device on the desk, and participants placed their right elbows on it to begin the VT (Fig. 2B). We did not place Cushion R until the beginning of the VT because participants had not used their right hand until the VT. We explained to participants that the purpose of using Cushion R was to prevent the pulse data from introducing noise from body movements when pressing the keyboard with the right hand during the task. Participants wore silicone earplugs in both ears to prevent loud noises from interfering with their thoughts.

**Figure 3:**
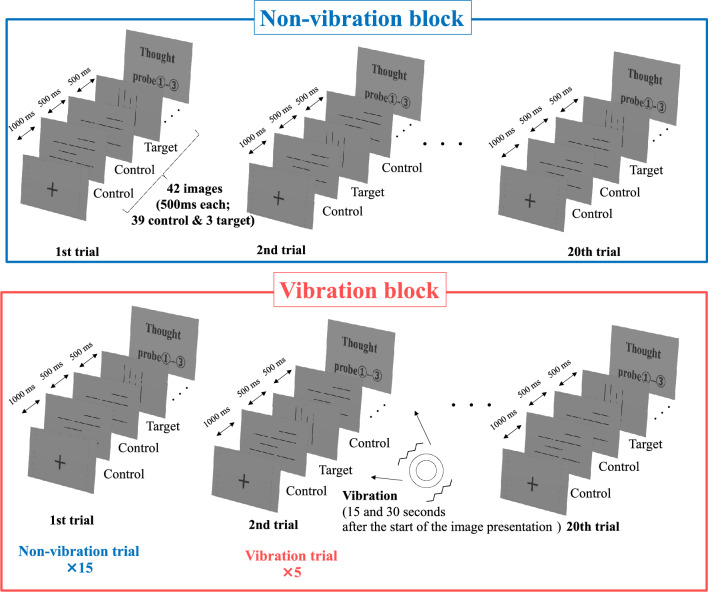
Timeline of the VT. Participants were asked to respond by pressing the space key only when presented with the target stimulus. We presented 42 image stimuli (3 of which were target stimuli) and thought probes per trial. If participants selected 1–5 in probe ①, probes ② and ③ were presented. If participants selected 6 in probe ①, probes ② and ③ were not presented, and participants proceeded to the next trial. Non-vibration block/vibration block included 20 trials each, and subliminal vibration stimuli were presented in five random trials of vibration block.

Participants performed a simple attention task for a sustained period, during which the shifting of their thought states was measured. First, a gazing cross was presented for 1s, and then 42 images (39 × control stimulus, 3 × target stimulus) and thought probes ①–③ were presented in one trial (Fig. 3). Examples of image stimuli are presented in Appendix Fig. A.1. The image stimulus presentation and inter-stimulus durations were 500 ms each. Participants responded by pressing the space key as soon as the target stimulus appeared on the screen. The time required for all image stimuli to be presented in one trial was 42 s, and no time limit was placed on responses to the thought probes. The order in which the target and control stimuli were presented in the trials was randomized trial-by-trial. The VT consisted of non-vibration block in the first half and vibration block in the second half, each block containing 20 trials. Participants took a 5-min break between the two blocks. We did not randomize the order of the blocks across participants to prevent the effects of the vibration presentation on heart rate from remaining in the later block (described in detail in “Heart rate variability (HRV)”). The vibration block consisted of 5 vibration trials and 15 non-vibration trials. In the vibration trials, a subthreshold vibration stimulus was presented 15 s and 30 s after the start of the image presentation. The order of vibration and non-vibration trials was random among participants, but the vibration trials were always arranged nonconsecutive. This was done to prevent participants from becoming aware of the vibration stimulus itself or their physical response to the stimulus, as the effect of the vibration stimulus is amplified by its successive presentation.

After the VT, the experimenter clarified that the vibration stimulus was presented later in the task and asked whether participants perceived the vibration stimuli themselves or a change in heart rate owing to some external manipulation. Participants who answered “yes” to this question were excluded from the analysis.

##### Thought categories

Three thought probes (Thought probe ①–③) were presented once per trial: thought content, contemplation (how deeply they thought), and consistency (coherence of thought contents). Since constraints on the transition from one mental state to another are important indicators in the framework of variation in thought states over time^1^, we estimated this from measures of how deep participants were stuck in a particular thought state (contemplation) and how scattered the content of their thoughts was (consistency). This frequency of probe presentation is generally consistent with the average duration of MW that has been self-caught in previous studies^70^. Because one block consisted of 20 trials, 20 combinations of data from thought probes ①–③ were obtained for each vibration block (non-vibration/vibration block). The number of thought probes presented per block was based on previous studies that measured thought content and its transition by probe-caught method, as in this study^71–74^.

In thought probe ①, participants reported their contents of thoughts immediately before the probe was displayed. We prepared the following six categories for participants to categorize their thoughts: Category 1, task-focused; Category 2, task-relevant (score of the task/elapsed time); Category 3, peripheral stimuli (changes in surrounding stimuli, sound/flash); Category 4, internal-body states (changes in bodily state, hunger/pain/heartbeat); Category 5, task-unrelated (past episodes, plans for the future); and Category 6, absent-mindedness (nothing). This classification was based on Stawarczyk et al.^68^, who distinguished the thoughts during the task in terms of task-relatedness (whether the thought contents were semantically related to the task itself) and stimulus-dependency (whether the thought contents were semantically related to the external and internal stimuli presented during the task). Category 1 was task-related and stimulus-dependent, Category 2 was task-related but stimulus-independent, Category 3 and 4 were task-unrelated but stimulus-dependent and Category 5 was task-unrelated and stimulus-independent. We set Category 6 as the option for participants to report if they could not monitor their thoughts. Category 1: task-focused is a state in which participants focused their thoughts on the performance improvement required in the VT (pressing the space key as soon as possible after the target stimulus is presented), and Category 2: task-relevant is a state in which their thoughts were related to the task such as elapsed time during the task or their own performance on the task, but not directly to the responses required in the VT. Since the purpose of this study was to discuss the role of interoception in thought shifting, as distinguished from exteroception (i.e., the perception of peripheral stimuli), we separated Categories 3 and 4. Additionally, we provided past episodes and future plans about oneself as specific examples of Category 5. This is based on previous research that showed that during MW, varius self-referential thoughts are reported, including simulations of one’s own past episodes, and future plans and goals^2,75^.

When participants pressed one of the number keys from 1 to 5 in thought probe ①, they responded to the contemplation of thoughts they answered in thought probe ① in thought probe ②, and to the consistency of thoughts in thought probe ③. However, for participants who answered 6 in thought probe ①, the next probes ② and ③ were not presented. In thought probe ②, participants responded by selecting one of the options ranging from “1. not very much” to “3. very deeply” to indicate how deeply they thought about the thoughts they responded to in probe ①. In thought probe ③, participants rated the degree to which they felt coherence between the contents of their immediate previous thoughts. Participants responded by pressing the number key 1, if they were thinking about a single matter, 2, if they were thinking about multiple contents and their semantic relevance was high, or 3, if their semantic relevance was low. We described in the Appendix the instructions we gave participants before the VT.

### Pre-processing

#### The HCT and the TET

We first performed pre-processing on the first task, the error rates for the HCT and the TET. We excluded data from two participants in our analysis of the HCT and the TET error rates. One participant was excluded because the heart rate data was not accurately measured in all tasks, and the other participant reported the same number of heartbeats in all conditions of the HCT and was considered to have not performed the task properly. Consequently, we used data from 98 university and graduate students (70 women, 28 men, *M*_*age*_ = 21 ± 1.50 years) to analyze both tasks. The HCT error rates for each participant were calculated using the following formula: ${1}/{6}\Sigma |{\text{actual heartbeats}}{-}{\text{reported heartbeats }}|/{\text{ actual heartbeats}}\, \times \,{1}00$^76^. The TET error rates for each participant were calculated using the following formula:${1}/{6}\Sigma |{\text{actual time lapse}}{-}{\text{reported time lapse}}|/{\text{ actual time lapse}}\, \times \,{1}00$. To confirm that the HCT results were not affected by inferences based on time estimates, we calculated Pearson’s product moment correlation coefficient, for the HCT and the TET error rates.

#### Effect on heart rate changes by vibration

We pre-processed the heart rate data before and after the presentation of vibration stimuli during the VT. At that time, data from 18 participants were excluded from the analysis, in addition to the two participants indicated in “The HCT and the TET”. Nine reported noticing vibration stimuli presented during the VT; three did not respond correctly on more than half of the trials of the VT; and six never reported any of 1, 2, 4, or 5 of thought probes ① in the entire VT. Consequently, we used data from 80 university and graduate students (54 women, 26 men, *M*_*age*_ = 21 ± 1.50 years) for the following analysis.

**Figure 4:**
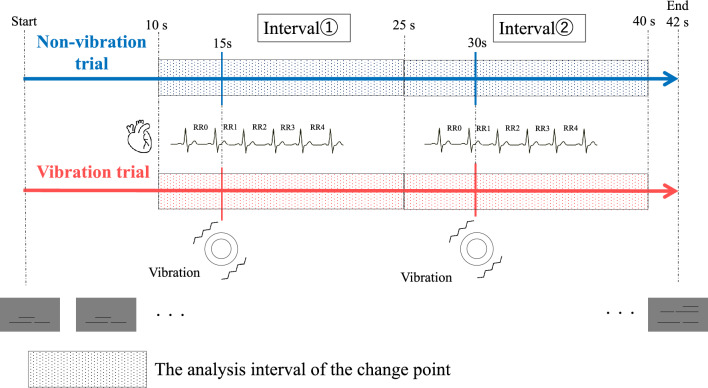
The timing of vibration presentation and the analysis interval of change point. We presented the vibration stimuli 15 and 30 s after the start of the trial in the vibration blocks. RR here is the time interval between the apexes of the pulse wave. RR0 is the RR interval immediately before the vibration, and RR1–4 are the RR intervals of the first, second, third, and fourth times after the vibration, respectively. In the non-vibration trials, which are just before the vibration trials, we defined RR0–4 based on 15 and 30 s after the start of the trial adjusting to the vibration trials. We analyzed change point detection for the RR interval from 5 s before to 10 s after the two vibration presentations in one trial (interval①, ②) and calculated the percentage of change points detected at RR1–3.

We examined whether the vibration presentation changed participants’ heart rate. The time interval at the apex of participant’s pulse wave (hereafter referred to as RR) was used as an index to track changes in participant’s heart rate. We presented the vibration stimuli 15 and 30 s after the start of the trial in the vibration trials (Fig. 4). The RR interval immediately before vibration presentation was defined as RR0, and the RR intervals at the first, second, third, and fourth times after vibration presentation were defined as RR1–4. The difference between RR1–4 respectively, and RR0 was calculated. Based on the trend of the difference between each time point and RR0, we considered that the vibration-induced changes in heart rate converged up to RR3, and after that, RR1–3 were considered in the analysis as the time points affected by the vibration presentation (Appendix Fig. A.2).

A change point analysis was performed using the R package “changepoint” (version 2.2.3) to compare the change in RR intervals when vibration stimuli were presented and when they were not^77^. We detected change points on vibration trials and on non-vibration trials that immediately preceded vibration trials. For the non-vibration trials, RR0–3 was defined as 15 and 30 s after the start of the trial. These two time points were when the vibration stimuli were presented in the vibration trial. The change points of the mean and variance of the RR intervals at 10–25 s (interval ①) and 25–40 s (interval ②) after the start of a trial were detected (Fig. 4). The reason we set the time window from 5 s before to 10 s after the stimulus presentation, is to examine the deviation from the vibration stimulus based on the trend of the heart rate immediately before and after the vibration presentation. The heart rate change rate for each block was calculated using the following equation:

$$\frac{{{\text{Total }}\;{\text{number}}\;{\text{ of }}\;{\text{times }}\;{\text{change}}\;{\text{ points }}\;{\text{were }}\;{\text{detected }}\;{\text{in RR}}\;{ }1,{ }2,{\text{ and}}\;{ }3}}{{{\text{The}} \;{\text{number }}\;{\text{of}}\;{\text{ applicable }}\;{\text{time points }}\left( {3;{\text{ RR}}1,2,3} \right) \times {\text{ The}}\;{\text{ number}}\;{\text{ of }}\;{\text{appropliate }}\;{\text{trials }}\;{\text{in }}\;{\text{each}}\;{\text{ block }}\left( {10} \right)}} \times 100$$

#### Thought probes

##### Thought probe ①–③

First, for each vibration/non-vibration block, we calculated the number of times each of the six categories of thought probe ① was reported (Appendix Fig. A.3). Because responses to Category 3: peripheral stimuli were significantly fewer than those to the other thoughts, we excluded them from subsequent analyses. We also excluded responses to Category 6: absent-mindedness from the analysis because they differed from the state of thought targeted in this study, in that the contents of the thoughts were not reported verbally.

Next, we calculated the rate of trials that responded with each of the options in thought probes ② and ③ out of the number of trials that responded with a response other than Category 6 in thought probe ① (Appendix Figs. A.4, A.5). Based on the distribution of responses and the expression of each option in thought probes ② and ③ (see “The VT”), we defined responses of 2 and 3 to probe ② as high contemplation and responses of 1 to probe ③ as high consistency. The specific distribution of responses to each option and their interpretation are described in the Appendix.

##### Indicators of thought states

We used two indices, transition, and continuation, as kinds of thought shifting. Transition refers to reporting different thought categories in thought probe ① on successive trials, and continuation refers to reporting the same thought category on successive trials. Because there is always interference by thought probes between trials (Fig. 3), continuation can be interpreted as a return to the same state of thought as before the external interference, rather than a state in which one continues to think about a particular matter all the time. Since one’s thoughts wandering to things unrelated to the task at hand is the general definition of MW^2^, we focused on the transition of thought between Category 1 in probe ① and the other categories (Categories 2, 4, and 5). We calculated transitions in two ways; one is to group all thoughts, except Category 1, into a single “MW state” and the other is to distinguish these states according to the content of the thoughts (see the Appendix). We calculated the number of times each transition/continuation pattern occurred for Categories 1, 2, 4, and 5. Moreover, additional analyses were conducted on contemplation and consistency in Category 5 because, as discussed in “Patterns of transition and continuation of thought”, specific effects of vibration presentation and interoceptive accuracy on thought shifting between trials were found only in Category 5. When Category 5 was reported in thought probe ①, we counted the number of times it met the high contemplation (selecting 2 or 3 in thought probe ②) or high consistency (selecting 1 in thought probe ③). We defined them as highly contemplated 5 and highly consistent 5, respectively.

#### Heart rate variability (HRV)

In this experiment, the vibration stimuli were presented in the latter block for all participants to avoid the possibility that the effect of the vibration-inducing heart rate change might remain in the subsequent block without the vibration presentation, and the “baseline” heart rate state unaffected by vibration might not be reproduced. However, since MW tends to increase with time^11^ and physical fatigue associated with the continuation of the task amplifies the frequency of MW^78,79^ and gives rise the conditions that can cause MW, such as depletion of attentional resources, negative emotions, and rumination^80^, it is possible that MW was more likely to occur in the second half of the task than in the first half. Therefore, by estimating autonomic nervous activity through heart rate data, we examined whether there was a significant difference in the physical condition between the first and second blocks. Using the fast Fourier transformation method, we quantified cardiovascular autonomic control from HRV. HRV data were collected and analyzed using Kubios HRV Standard 3.6.5^81^. The following parameters are mainly used; very low frequency, < 0.003–0.04 Hz (VLF), low-frequency power, 0.04–0.15 Hz (LF), high-frequency power, 0.15–0.4 Hz (HF), and the ratio LF/HF. We featured HF and LF/HF, the former is often understood as a representation of the parasympathetic nervous system, and the latter might reflect the global sympathetic/vagal balance^82^. We calculated HF and LF/HF power values for each participant for the last 3/4 of each block and compared these values across blocks.

### Modeling

We used Bayesian approach for estimating parameters in the statistical model. This is because we would like to stabilize convergence in estimating models with many parameters. There has been an increasing number of attempts to analyze self-reported data on thought using Bayesian statistical modeling^83,84^. We used a generalized linear mixed model to account for the distributional characteristics of each acquired dataset. The brms R package was used for the analysis^85,86^, and parameters were estimated with four Markov Chain Monte Carlo chains. Each chain contained 1000 burn-in samples and 2000 additional samples with a thinning parameter of 1, resulting in 1000 posterior samples per chain, that were combined into one posterior sample consisting of 4000 samples for each model parameter. Regression weights had Cauchy distributed priors, *b*_*i*>*0*_ ∼ *N* (0, 3), and intercepts, while standard deviations of random effects had Student’s *t*-distributed priors, *b*_*0*_ ∼*t* (3, 0, 2.5). Model convergence was evaluated based on the Gelman–Rubin convergence statistic *R̂*^87^, with it close to 1 indicating negligible differences between within- and between-chain variances. We report the mean and 95% credible interval (CI) as an equally tailed interval, to describe the posterior distributions of sampled regression weights. To account for individual differences of each index, they were set as a random intercept for all modeling.

#### Induction of heart rate changes

To investigate whether there is a difference in the heart rate change rate between the non-vibration trials and the vibration trials, we used Bayesian generalized mixed models, assuming a Gaussian distribution with an identity link function, to accommodate for the distributional properties of the heart rate change rate. We included the main effect of vibration (0 = non-vibration, 1 = vibration), participants’ HCT error rates (= score), and participants’ sex (0 = men, 1 = women). To examine the effect of vibration on heart rate, we focused on the coefficient of the main effect of vibration.

#### Thought characteristics

To investigate how the patterns of thought transition and continuation, as well as the contemplation and consistency of Category 5, differ depending on vibration and the accuracy of interoception, we used Bayesian generalized mixed models assuming a Poisson distribution with a logit link function, to accommodate for the distributional properties of the number of the reports of each thought conditions. For all modeling, we included the main effects of participants’ error rates of the HCT (= score), vibration (0 = non-vibration, 1 = vibration), and participants’ sex (0 = men, 1 = women). Moreover, we included interaction of participants’ HCT and vibration error rates (= score: vibration). To examine how the relationship between interoceptive accuracy and the presence or absence of vibration affects the characteristics of thought, we focused on the coefficient of interaction between the HCT error rates and vibration.

#### HRV

To investigate whether there is a difference in the power value of HRV parameters between the non-vibration block and the vibration block, we used Bayesian generalized mixed models assuming a Gaussian distribution with an identity link function, to accommodate for the distributional properties of the power value of HF and LF/HF. We included the main effects of vibration (0 = non-vibration, 1 = vibration), and participants’ sex (0 = men, 1 = women). To examine the effect of different blocks of the VT; that is, vibration conditions, on the autonomic function of participants, we focused on the coefficient of the main effect of vibration.

## Results

### The HCT and the TET

**Figure 5:**
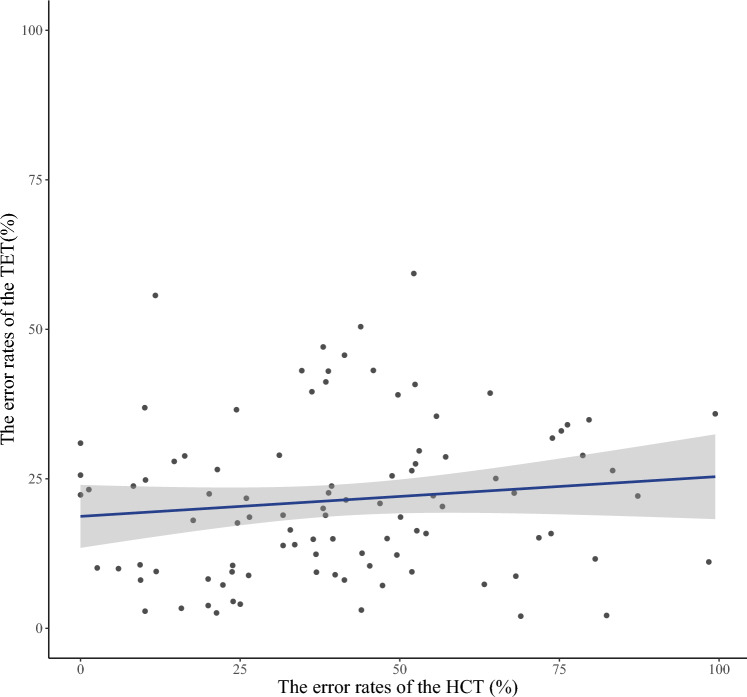
The relationship between the error rates of the HCT and the TET. The vertical and horizontal axes represent the HCT and the TET error rates respectively. The dots indicate the measured error rates. The solid line is the regression line. There was little correlation between the two error rates.

The mean error rate for the HCT was 40.69 with a standard deviation of 23.48. The mean error rate for the TET was 21.44 with a standard deviation of 13.10. There was little correlation between the error rates of the HCT and that of the TET (Fig. 5, *r*_98_ = 0.11, *p* = 0.24). Because these results suggest that the performance of HCT was not affected by the time-estimated inferences, the error rates of the HCT were used as a measure of interoceptive accuracy in this later analysis.

### Induction of heart rate changes

**Figure 6:**
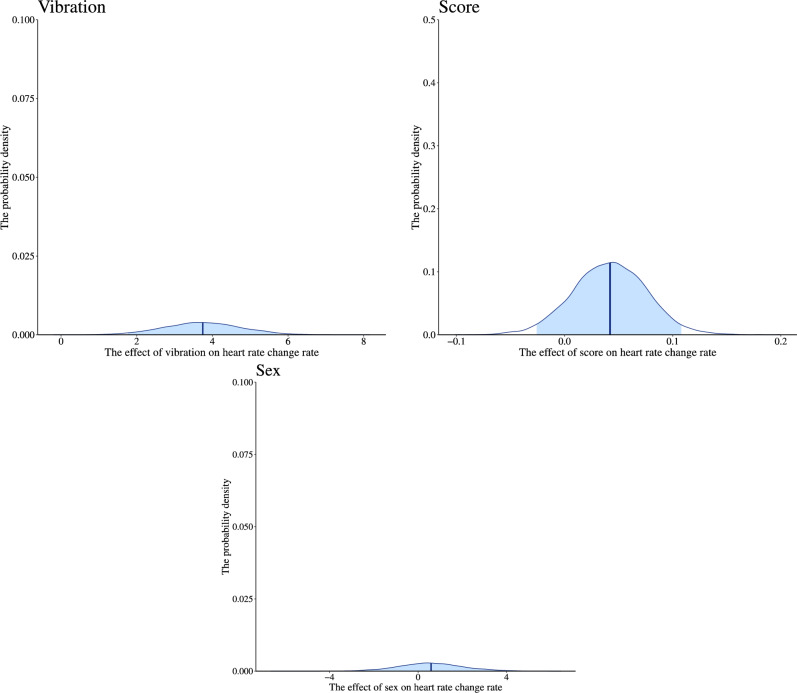
Distribution of the estimated parameters of heart rate change rate. We indicated the influence of each factor on the heart rate change rate on the horizontal axis and the probability density on the vertical axis. The dark blue line shows the estimated coefficient, and the light blue range shows the 95% credible interval (CI). The lower limit of the 95% CI in main effect of vibration (0 = non-vibration, 1 = vibration) is higher than 0, indicating that this factor influences the heart rate change rate. The 95% CI of main effect of score and sex includes 0, indicating that this factor has little effect on the change rates of heart rate.

**Figure 7:**
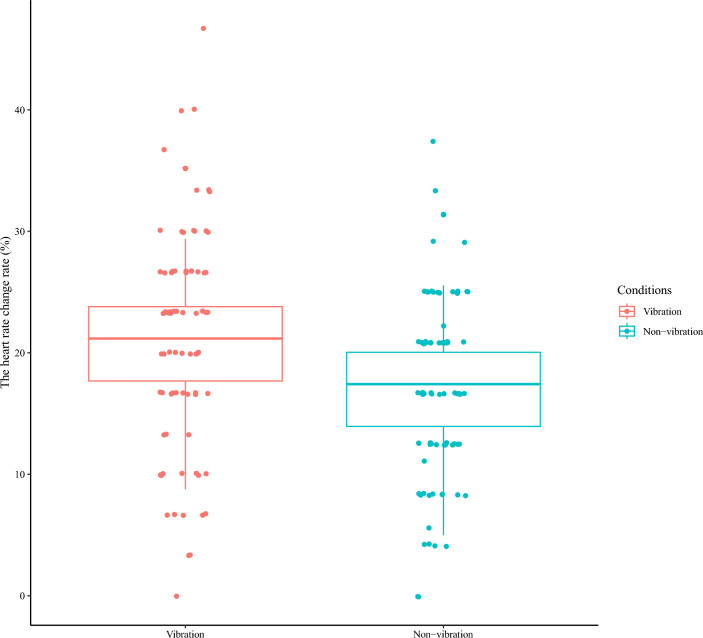
The posterior predictive distribution of the probability of detecting a change point at RR1–3 for each condition. We show the condition name (vibration/non-vibration) on the horizontal axis and the estimated heart rate change rate on the vertical axis. The points on the graph indicate measured values. RR0 is the RR interval immediately before vibration; RR1–3 are the first, second, and third RR intervals after vibration. Change point detection was analyzed for the RR interval from 5 s before to 10 s after each vibration presentation, and the percentage of change points detected at RR1, 2, or 3 was calculated. The probability of detecting the change points at RR1–3 was higher in the vibration block than in the non-vibration block, suggesting that this experiment's heart rate change induction was successful.

To investigate whether subliminal vibration increases heart rate change rate in the vibration block, we performed the modeling described in “Induction of heart rate changes”. All chains converged for the model, as indicated by *R̂* equal to 1. We found the main effect of vibration, *b* = 3.734, 95% CI [1766; 5.644]. Contrastingly, there was little effect of score and sex (Table 1, and Fig. 6). The heart rate change rate was higher for the vibration block (Fig. 7). The results show that more changes in heart rate occurred immediately after the presentation of the subliminal vibration stimuli compared to the same time point in the condition without it, and that this effect was independent of participants’ interoceptive accuracy or sex. We concluded that the heart rate change induction in this experiment was successful.

**Table 1:** Summary of estimated parameters of heart rate changes.

Parameters	Estimate	Est. error	l-95% CI	u-95% CI	*R̂*	Bulk_ESS	Tail_ESS
Intercept	14.905	1.900	11.216	18.733	1	3034	3001
Vibration	3.734	0.992	1.766	5.644	1	8677	2640
Score	0.042	0.034	− 0.026	0.108	1	2830	2696
Sex	0.836	1.533	− 2.104	3.943	1	3638	2971

Notes:

Since *R̂* = 1 for all parameters, the model has converged; Estimate: the estimated value of each parameter; Est. Error: the error in the estimated value of each parameter; l-95% CI: the lower limit of the 95% credible interval (CI); u-95% CI: the upper limit of the 95% CI; *R̂*: indicators for determining convergence of Markov Chain Monte Carlo (MCMC) chain; Usually, the model is considered to have converged at *R̂* ≤ 1.1.; Bulk_ESS: the effective sample size calculated by normal rank; Tail_ESS: the effective sample size for the 5% and 95% quantile points; Vibration: the main effect of with or without vibration presentation (0 = non-vibration, 1 = vibration); Score: the main effect of participants’ error rates of the HCT; Sex: participants’ biological sex.

### Patterns of transition and continuation of thought

First, to examine whether participants’ interoceptive accuracy and the presence or absence of unconscious deviation of heart rate would affect thought transition, we performed the modeling described in “Thought characteristics” for the transition of thought (see “Indicators of thought states”). There was no interaction between score and vibration in any of these transition patterns (Appendix Table A.1–8 and Fig. A.6–8). The results indicate that the presence or absence of unconscious heartbeat deviation, interoceptive accuracy, or their interaction do not affect the transition between the task-focused state and other thoughts as defined in this experiment.

**Figure 8:**
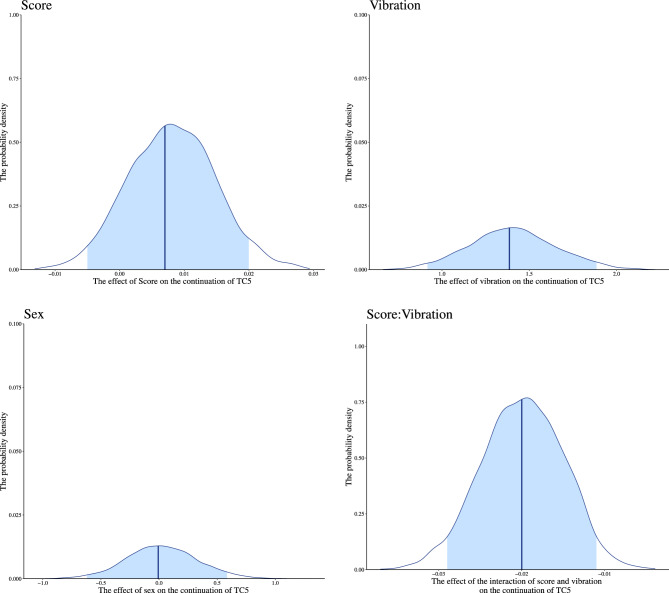
Distribution of each estimated parameter in the continuation of TC5. We indicated the influence of each factor on the continuation of TC5 on the horizontal axis and the probability density on the vertical axis. The dark blue line shows the estimated coefficient, and the light blue range shows the 95% credible interval (CI). The lower limit of the 95% CI in main effect of vibration is higher than 0, and the higher limit in interaction of score and vibration is lower than 0. This indicates that these factors influence the continuation of TC5. The 95% CI of main effect of the HCT error rates and sex includes 0, indicating that these factors have little effect on the continuation of TC5.

**Figure 9:**
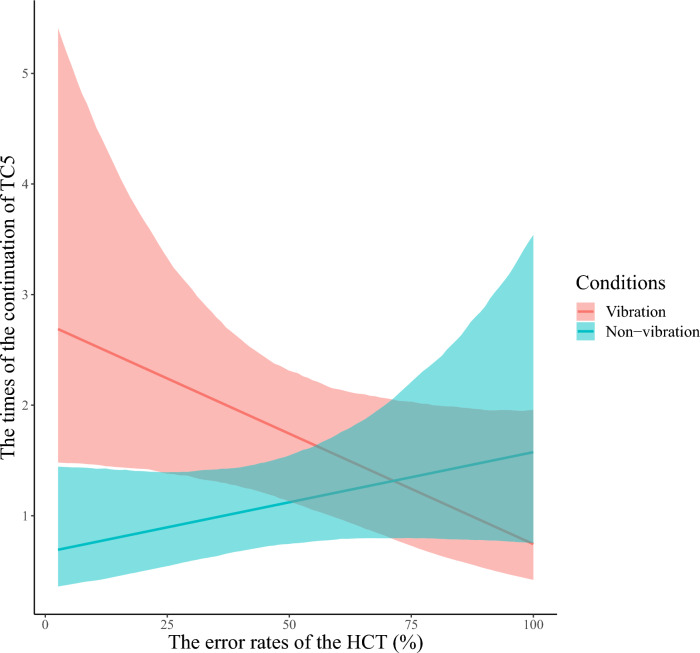
The posterior predictive distribution of the relationship between the HCT error rates and the number of reported continuation of TC5 for each vibration condition. TC5 are reports of self-referential thoughts, such as past episodes and future plans regarding oneself. We show the HCT error rates on the horizontal axis and the estimated number of the continuation of TC5 reports on the vertical axis. The colors indicate the conditions (vibration/non-vibration). The solid line on the graph shows the regression line based on the estimated values. Each colored range indicates 95% CI. Participants with the lower HCT error rates; that is, more accurate interoception, reported a greater number of the continuation of TC5 in the vibration block compared to the non-vibration block. Contrastingly, the lower the interoceptive accuracy, the smaller the difference in the frequency of reports of the continuation of TC5 between vibration conditions.

Second, to examine whether participants’ interoceptive accuracy and the presence or absence of unconscious deviation of heart rate would cause a difference in the continuation of thought, we performed the modeling described in “Thought characteristics” for the continuation of thought Category 1, 2, 4, and 5 (see “Indicators of thought states”). For the continuation of thought Category 5: task-unrelated (hereafter, referred to as TC5), all chains converged for the model, as indicated by *R̂* equal to 1. We found the main effect of vibration and interaction between score and vibration *b* = -0.020, 95% CI [− 0.029; − 0.011], for the continuation of TC5. Contrastingly, there was little effect of sex and score (Table 2 and Fig. 8). The higher the accuracy of interoception, the higher the number of reported continuation of TC5 in the vibration block (Fig. 9). There was no interaction between interoceptive accuracy and vibration for the continuation of thoughts other than TC5 (Appendix Table A.9–11, and Fig. A.9). When unconscious deviations in heartbeat occur, the greater the ability to accurately capture them, the more likely the TC5; that is, task-unrelated and self-referential thoughts (see “Thought categories”), would continue. No similar results were found for the other categories of thought, suggesting that changes in bodily response and perception of them specifically affect TC5.

**Table 2:** Summary of estimated parameters of the continuation of TC5.

Parameters	Estimate	Est. error	l-95% CI	u-95% CI	*R̂*	Bulk_ESS	Tail_ESS
Intercept	− 0.240	0.351	− 0.932	0.436	1	1068	1791
Score	0.007	0.006	− 0.005	0.020	1	1156	1774
Vibration	1.387	0.246	0.916	1.886	1	2493	2795
Sex	− 0.007	0.300	− 0.621	0.586	1	863	1568
Score: Vibration	− 0.020	0.005	− 0.029	− 0.011	1	2602	2941

Notes:

Since *R̂* = 1 for all parameters, the model has converged; Estimate: the estimated value of each parameter; Est. error: the error in the estimated value of each parameter; l-95% CI: the lower limit of the 95% credible interval (CI); u-95% CI: the upper limit of the 95% CI; *R̂*: indicators for determining convergence of Markov Chain Monte Carlo (MCMC) chains; Usually, the model is considered to have converged at *R̂* ≤ 1.1.; Bulk_ESS: the effective sample size calculated by normal rank; Tail_ESS: the effective sample size for the 5% and 95% quantile points; Score: main effect of participants’ error rates of the HCT; Vibration: main effect of with or without vibration presentation (0 = non-vibration, 1 = vibration). Sex: participants’ biological sex; Score/Vibration: interaction of participants’ error rates of the HCT and with or without vibration presentation.

### Post-hoc analysis of contemplation and consistency of category 5

**Figure 10:**
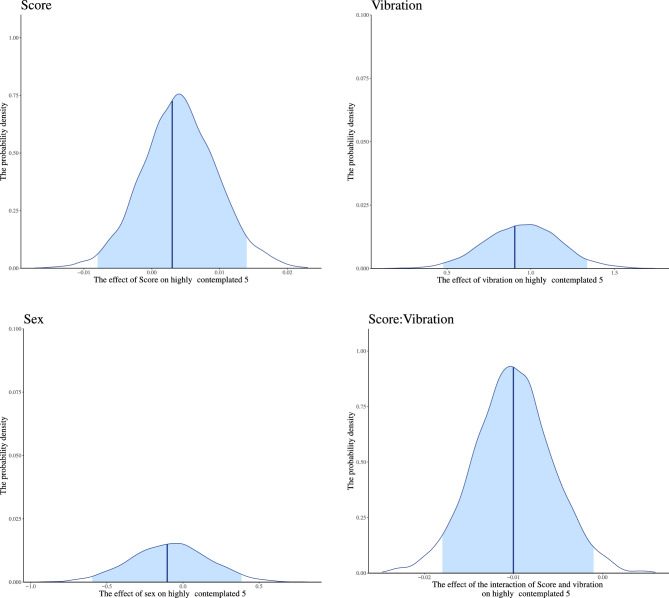
Distribution of each estimated parameter in highly contemplated 5. We indicated the influence of each factor on highly contemplated 5 on the horizontal axis and the probability density on the vertical axis. The dark blue line shows the estimated coefficient, and the light blue range shows the 95% credible interval (CI). The lower limit of the 95% CI in main effect of vibration is higher than 0, and the higher limit in interaction of the error rates of the HCT and vibration is lower than 0. This indicates that these factors influence highly contemplated 5. The 95% CI of main effect of the HCT error rates and sex includes 0, indicating that these factors have little effect on highly contemplated 5.

**Figure 11:**
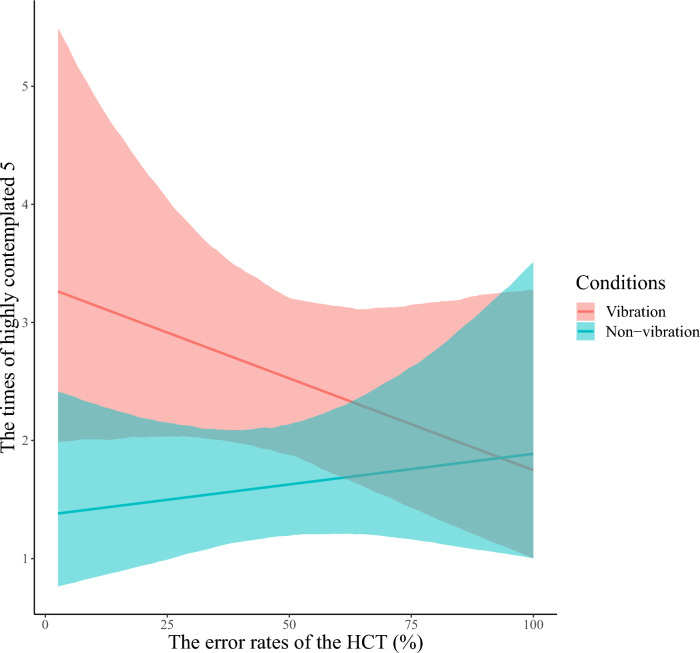
The posterior predictive distribution of the relationship between the HCT error rates and the number of reported highly contemplated 5 for each vibration condition. We show the HCT error rates on the horizontal axis and the estimated number of highly contemplated 5 reports on the vertical axis. The colors indicate the conditions (vibration/non-vibration). The solid line on the graph shows the regression line based on the estimated values. Each colored range indicates 95% CI. Participants with the lower HCT error rates; that is, more accurate interoception, reported more highly contemplated 5 in the vibration block compared to the non-vibration block. Contrastingly, the lower the interoceptive accuracy, the smaller the difference in the frequency of reports of highly contemplated 5 between vibration conditions.

Since we found an interaction between interoceptive accuracy and vibration in the continuation of TC5 in section “Patterns of Transition and Continuation of Thought”, we examined whether there is an interaction between interoceptive accuracy and vibration in contemplation and consistency of TC5. We performed the modeling described in “Thought characteristics” for highly contemplated and highly consistent 5 (see “Indicators of thought states”). For highly contemplated 5, all chains converged for the model, as indicated by *R̂* equal to 1. We found the main effect of vibration, and interaction between score and vibration *b* = − 0.010, 95% CI [− 0.018; − 0.001], for highly contemplated 5 (Table 3, and Fig. 10). The higher the interoceptive accuracy, the more frequently a highly contemplated TC5 is reported in the vibration block (Fig. 11). There was no interaction between interoceptive accuracy and vibration for highly consistent 5 (Appendix Table A.12 and Fig. A.10). These results indicate that the greater the ability to capture unconscious deviations in bodily response accurately, the more likely one is to contemplate TC5.

**Table 3:** Summary of estimated parameters of highly contemplated 5.

Parameters	Estimate	Est. error	l-95% CI	u-95% CI	*R̂*	Bulk_ESS	Tail_ESS
Intercept	0.316	0.304	− 0.306	0.896	1	1060	1700
Score	0.003	0.006	− 0.008	0.014	1	1124	2206
Vibration	0.904	0.219	0.476	1.338	1	2977	2902
Sex	-0.103	0.253	− 0.596	0.385	1	957	1925
Score: Vibration	-0.010	0.004	− 0.018	− 0.001	1	2788	3073

Notes:

Since *R̂* = 1 for all parameters, the model has converged; Estimate: the estimated value of each parameter; Est. error: the error in the estimated value of each parameter; l-95% CI: the lower limit of the 95% credible interval (CI); u-95% CI: the upper limit of the 95% CI; *R̂*: indicators for determining convergence of Markov Chain Monte Carlo (MCMC) chains; Usually, the model is considered to have converged at *R̂* ≤ 1.1.; Bulk_ESS: the effective sample size calculated by normal rank; Tail_ESS: the effective sample size for the 5% and 95% quantile points; Score: main effect of participants’ error rates of the HCT; Vibration: main effect of with or without vibration presentation (0 = non-vibration, 1 = vibration); Sex: participants’ biological sex; Score/Vibration: interaction of participants’ error rates of the HCT and with or without vibration presentation.

### HRV

To examine whether there were any differences in the physical condition of participants between the first and second halves of the experiment, we performed the modeling described in “HRV” for HF and LF/HF values. For both values, all chains converged for the models, as indicated by *R̂* equal to 1. We found no main effects of block for both models (Appendix Table A.13 and 14 and Fig. A.11). Therefore, it can be considered that the passage of time owing to the continuation of the task did not affect participants’ physical condition.

## Discussion

The study aimed to examine the effects of unconscious changes in bodily response and the individual’s interoceptive accuracy on thought shifting. This study hypothesized that (1) changes in heart rate by vibration would induce thought shifting, and (2) the effect of (1) would depend on one’s interoceptive accuracy. We measured participants’ interoceptive accuracy by the HCT and their thought state by presenting thought probes during the VT. Additionally, we induced unconscious changes in participants’ heart rate by presenting subliminal vibration stimuli during the VT.

To test the above hypothesis, we examined participants’ interoceptive accuracy and the effect of the heart rate change induction by vibration on the characteristics of their thought states (see “Indicators of thought states” and “Thought characteristics”). We found an interaction between interoceptive accuracy and vibration, in addition to the main effect of vibration, for the continuation and contemplation of self-referential MW (TC5). Continuation in this experiment refers to reporting the same category of thought content on successive trials, and contemplation measures how deeply participants thought about the reported thought content. Self-referential MW is a state of thinking about past episodes or future plans concerning the self, among MW that is thought unrelated to the task at hand. Moreover, there was little effect of interoceptive accuracy alone for either continuation or contemplation of self-referential MW (TC5; Tables 2 and 3; Figs. 8 and 10). Posterior predictive distribution showed that when the vibration-induced unconscious heart rate changes occurred, the more accurate one’s interoception was, the more self-referential MW (TC5) was continued and the higher its contemplation was. (Figs. 9 and 11). These results suggest that people with accurate interoception are not unique in how self-referential MW (TC5) arises; rather, they accurately capture larger-than-normal changes in heart rate, enhancing the continuation of self-referential MW (TC5) and its contemplation.

Notably, the “continuation” of thought as measured in this experiment is not necessarily the same as goal-directed thought—the state of continuing to think about a particular matter^1^. We referred to the continuation of thought when participants reported that they were in the same thought state in successive trials. We collected these thoughts using the probe-caught method, in which probe stimuli are randomly presented during the task and participants respond to their immediate prior conscious experience^88^. Because participants were asked to retrospectively evaluate not only the content of their immediate previous thought but also its contemplation and consistency during the probe response, it is likely that the presentation of the probe interrupted their thought and shifted them into a state of meta-consideration of it. Although the same thought appears to be continuing in the data, what was actually occurring in the continuation was a process in which a certain thought was interrupted by the probe and a meta-evaluation of that thought was made, and then a return to the same state of thought as before the probe was presented. That is to say, cardiovascular responses to subliminal vibration stimuli specifically produce intermittent regression to self-referential MW (TC5). These results suggest that for specific MW whose content is related to the self, the interoceptive information processed subconsciously triggers MW as well as the exteroceptive stimuli^3,12–15^, and this effect is stronger in individuals with accurate interoception.

In these terms, the present results partially support the hypothesis. The contribution of interoception to MW awareness has been elucidated^39,40^; however, the current results suggest that interoception may also be involved in the process of shifting to a specific thought. This view is consistent with previous studies that suggested that limbic areas, including the posterior insula, involved in the representation of interoceptive information^35,36^ are activated during the generation of spontaneous thought, and that this might be owing to changes in the saliency of the internal environment, such as memory recall and the perception of emotional/physical states^89^. The sensory and emotional saliency associated with such somatosensory processing could automatically limit thought states independent of one’s intentions and increase the contemplation of thought^1,90^. The higher the accuracy of interoception based on the HCT scores, the higher the connectivity between posterior insular cortex, which is responsible for processing such saliency, and the saliency network^66^. Consequently, it is possible that participants with accurate interoception in this study detected some degree of saliency, even in the case of weak, unconscious changes in their bodily response, resulting in a higher frequency of highly contemplative self-referential MW (TC5).

The interoceptive inference theory could explain why this effect of interoception on shifting thoughts is found only specifically in self-referential MW (TC5;^91^). This theory states that our brains have top-down predictions about interoceptive inputs, and that the process of minimizing the prediction error between the actual interoceptive input and the predicted one gives rise to concepts of self-related to the perception of emotion and the experience of body ownership. In our experiment, the subliminal vibration stimulation could have caused a change in heart rate, resulting in a prediction error between the prediction of interoceptive input and the actual input. This theory suggests that the prediction error minimization of interoception could be related not only to the low-level self-concept of the experience of body ownership but also to higher-level self-concepts such as first-person perspective, intention, and subjectivity^91,92^, and that self-related thought could have occurred intermittently in the process of minimizing the prediction error caused by the heartbeat change. Contrastingly, some body information always influences neural processing even without specific body changes, and this information could generate self-related thoughts^37^. In the present experiment, self-referential MW (TC5) was more likely to continue when vibration-induced heart rate changes occurred; however, it is possible that the default state of body information processing also had an influence here. In the future, we plan to develop a method of heartbeat change induction so that the degree of heartbeat deviation can be manipulated in more detail, and then measure neural activity during heartbeat manipulation to determine (1) what level of body change triggers the prediction error minimization process of interoception centered in the anterior insular cortex^91^, and (2) whether the subjective nature (content, emotional valence, vividness, etc.) of the self-related thoughts differ between cases in which the prediction error minimization process is triggered by a heartbeat change and cases in which no specific physical change is present.

One of the most novel aspects of this study was the attempt to induce changes in heart rate by presenting subliminal vibration stimuli. We found the main effect of with or without vibration on the heart rate change rate (Table 1 and Fig. 6). The distribution of the posterior predicted detection rate of change points showed that the heart rate change rate was higher for trials in which vibration was presented (Fig. 7). In this experiment, participants who noticed the vibration presentation itself or the changes in heart rate caused by it were excluded from the analysis (9 out of 100 participants). Given this, the results indicate that presenting subliminal vibration stimuli to a part of the body can produce larger-than-normal heart rate fluctuations in unconsciousness. To our knowledge, studies have yet to demonstrate that changes in heart rate can be induced without disclosing the possibility of stimulus presentation to participants. While previous studies found that the cardiac cycle influences the detection of near-threshold tactile stimuli^62,63^, the current results suggest that subliminal tactile stimulation alters cardiac activity. Future studies should examine changes in stimulus detectability owing to the interaction between subliminal vibration stimulus presentation and cardiac activity. We find it very interesting that the heart rate change rate in this experiment was not affected by the error rates of the HCT (i.e., one’s interoceptive accuracy). This suggests that interoception is only involved in signal processing from inside the body^19,20^, and that different factors, such as individual stimulus thresholds and body size, are relevant to how external stimuli affect bodily responses.

This study had some limitations. First, we could not measure the threshold for each participant in the subliminal vibration presentation. The “subliminal” in this study is merely that participants reported that they did not feel any vibration in their retrospective verbal reports after the experiment. We are currently conducting an experiment in which we measure participants’ thresholds using other stimulus devices, present subliminal stimuli, and measure changes in physiological responses. Examining whether more accurate induction of heart rate changes is possible based on the results of this experiment is necessary. Second, the thought measurement paradigm used in this experiment may not capture minute changes in thought. We used a probe-caught method, in which participants reported their thought states only at timings set by the experimenter (once every 42 s). In studies in which thought probes were presented more frequently than in the present experiment (^93^ (once every 25 s to 175 s)^83^, (once every 30 s or 60 s)), the rate of MW reporting decreases as the frequency of probe presentation increases. However, since the frequency of probing did not affect the performance of the sequential reaction tasks performed in parallel (this is known to decrease with MW), it is suggested that the frequency of probing, rather than the actual frequency of MW occurrence itself or the reaction to probing, could be causing participants' resistance to reporting MW by prompting frequent awareness of MW^94^. Contrastingly, as already mentioned, in a study using the self-caught method, MW was reported at a frequency similar to the probe interval in this experiment^70^. Consequently, the current experiment may capture natural thought shifting, and we believe that changing the frequency of presentation of the thought probes in this experiment would not affect the results reported here. However, when considering individual thought states, there is a possibility that transitions in thought occur between the previous thought probe and the current one, and it is assumed that our method does not reflect all these transitions. Developing a paradigm that can reproduce more natural transitions in thought, such as manipulating the timing of probe presentation based on objective indices that are thought to reflect transitions in thought states, (e.g., reaction time to a task and eye movements), is necessary.

This study was an exploratory investigation of the direct relationship between interoceptive processing and thought shifting. The novelty of this study is that it showed the following three points: (1) the presentation of subliminal vibration can induce changes in physiological responses, (2) changes in interoception specifically trigger MW related to the self, and (3) the effect of interoception on thought transitions depends on interoceptive accuracy based on cardiac perception. The paradigm contributes to future research on thought by presenting a methodology for examining the effects of internal-body responses induced by subliminal external stimuli on thought states. Researchers should examine the relationship between the processing of bodily response and thought in more detail by strictly controlling subliminal stimuli, comprehensively examining factors other than interoception that influence thought shifting, and measuring brain activity during the task.

## Conclusion

This study examined the effects of unconscious changes in bodily response and individuals’ interoceptive accuracy on thought shifting. For people with high interoceptive accuracy, when subliminal vibration stimuli induced unconscious changes in heart rate, self-referential thought occurred intermittently, and the contemplation of these thoughts was enhanced. This study is significant because it showed the possibility that bodily response and accurate perception of it could influence shifting toward a particular thought state.

### Supplementary Information

Supplementary Information.

## Data Availability

The data that support the current findings are available on request from the corresponding author (M.S.). The data are not publicly available because we did not obtain consent from participants.
