# Supplementary material for: Effect of subconscious changes in bodily response on thought shifting in people with accurate interoception
Source: Sci Rep. 2023 Oct 3;13:16651. doi: 10.1038/s41598-023-43861-w (PMC10547779; doi:10.1038/s41598-023-43861-w)
Supplement: Supplementary file 1 — Supplementary Information. [file 41598_2023_43861_MOESM1_ESM.docx]

# Appendix. Supplementary data

**Fig. A.1.** Examples of image stimuli used in VT.

**Fig. A.2.** The time series of the difference between each of RR1,2,3,4 and RR0. RR here is the time interval between the apexes of the pulse wave. The RR interval immediately prior to vibration presentation was defined as RR0, and the RR intervals at the first, second, third, and fourth times after vibration presentation were defined as RR1–4. Error bars represent standard errors. We assumed that a large change in the RR interval occurred at RR3, after which the changes converged.

**Thought Probe ①–③**

To assess the degree of contemplation and consistency of thought for each of the categories of thought answered in thought probe ①, the high/low categories of contemplation and consistency were determined based on the distribution of responses to each option. In probe ②, the reporting rate was higher for 1, 2 and 3, in that order, for both vibration conditions; thus, the response to 1 was designated as low contemplation and the responses to 2 and 3 as high contemplation, considering the expressions in the options (2.3.2.2.). In probe ③, the reporting rate of 1 was the highest, and there was little difference between those of 2 and 3 for both vibration conditions; thus, the response to 1 was designated as high-consistency and the responses to 2 and 3 as low-consistency, considering the expressions in the options (2.3.2.2.).

Fig. A.3. Distribution of the number of times each thought included in thought probe ① was reported. The points on the graph indicate the measured number of reports. Error bars represent standard errors. We excluded Category 3: peripheral stimuli from subsequent analyses since it was clearly reported less frequently than the other thought contents. For Category 5:task-unrelated, the number of reports was distributed at a slightly higher value for the vibration block compared to the non-vibration block. Task-unrelated are reports of self-referential thoughts, such as past episodes and future plans regarding oneself.

Fig. A.4. The reporting rates for each option of thought probe ②. Error bars represent standard errors. The points on the graph indicate the measured reporting rates. In probe ②, participants responded by selecting one of the options from “1. not very much” to “3. very deeply” to indicate how deeply they contemplated the thoughts they responded to in probe ①.

Fig. A.5. The reporting rates for each option of thought probe ③. Error bars represent standard errors. The points on the graph indicate the measured reporting rates. In probe ③, participants responded by pressing the number key 1, if they were thinking about a single matter, 2, if they were thinking about multiple contents and their semantic relevance was high, or 3, if their semantic relevance was low.

**Results**

We present here the results of the modeling that we were unable to include in the text. We show the distribution of the estimated values of each parameter in the tables. Further, we show the distribution of estimated parameters about the coefficient of interaction between the HCT error rates and vibration in the figures. In the figures, the dark blue line shows the estimated coefficient, and the light blue range shows the 95% credible interval (CI). In all modeling, the 95% CI of the estimated coefficients of interaction of error rates of the HCT and vibration includes 0, indicating that there was little effect of the interaction between the HCT error rates and vibration for each phenomenon. From this point on, we use “TC+number” for each category of thought defined in thought probe ①.

**Table A.1**

Summary of estimated parameters of the transition of thought from TC1 to MW state (TC2,4,5).

| Parameters | Estimate | | Est. error | l-95% CI | u-95% CI | *R̂* | Bulk_ESS | Tail_ESS |
| --- | --- | --- | --- | --- | --- | --- | --- | --- |
| Intercept | 0.812 | | 0.210 | 0.401 | 1.212 | 1 | 3163 | 3134 |
| Score | -0.003 | | 0.004 | -0.011 | 0.005 | 1 | 3084 | 2980 |
| Vibration | -0.348 | | 0.225 | -0.779 | 0.097 | 1 | 3288 | 2789 |
| Sex | 0.182 | | 0.174 | -0.156 | 0.527 | 1 | 2953 | 2887 |
| Score:Vibration | | 0.004 | 0.004 | -0.005 | 0.013 | 1 | 3316 | 2870 |

**Table A.2**

Summary of estimated parameters of the transition of thought from MW state (TC2,4,5) to TC1.

| Parameters | Estimate | | Est. error | l-95% CI | u-95% CI | Rhat | Bulk_ESS | Tail_ESS |
| --- | --- | --- | --- | --- | --- | --- | --- | --- |
| Intercept | | 0.585 | 0.264 | 0.050 | 1.102 | 1 | 2642 | 2828 |
| Score | | -0.005 | 0.005 | -0.014 | 0.005 | 1 | 2880 | 3032 |
| Vibration | | -0.445 | 0.250 | -0.937 | 0.050 | 1 | 4061 | 2715 |
| Sex | | 0.306 | 0.227 | -0.134 | 0.761 | 1 | 2110 | 2710 |
| Score:Vibration | | 0.005 | 0.005 | -0.005 | 0.015 | 1 | 4040 | 3055 |

**Fig. A.6.**

**Table A.3**

Summary of estimated parameters of the transition of thought from TC1 to TC2.

| Parameters | Estimate | Est. error | l-95% CI | u-95% CI | *R̂* | Bulk_ESS | Tail_ESS |
| --- | --- | --- | --- | --- | --- | --- | --- |
| Intercept | 0.055 | 0.295 | -0.536 | 0.606 | 1 | 2880 | 3278 |
| Score | -0.003 | 0.006 | -0.014 | 0.008 | 1 | 2887 | 2917 |
| Vibration | -0.655 | 0.336 | -1.328 | -0.009 | 1 | 3025 | 3127 |
| Sex | 0.290 | 0.240 | -0.186 | 0.756 | 1 | 3351 | 2974 |
| Score:Vibration | 0.008 | 0.007 | -0.005 | 0.021 | 1 | 3223 | 2561 |

**Table A.4**

Summary of estimated parameters of the transition of thought from TC1 to TC4.

| Parameters | Estimate | Est. error | l-95% CI | u-95% CI | *R̂* | Bulk_ESS | Tail_ESS |
| --- | --- | --- | --- | --- | --- | --- | --- |
| Intercept | -0.586 | 0.390 | -1.368 | 0.139 | 1 | 2768 | 2836 |
| Score | -0.001 | 0.007 | -0.015 | 0.012 | 1 | 2821 | 3042 |
| Vibration | -0.547 | 0.491 | -1.503 | 0.411 | 1 | 3284 | 3169 |
| Sex | -0.382 | 0.329 | -1.059 | 0.249 | 1 | 4035 | 2514 |
| Score:Vibration | 0.005 | 0.009 | -0.013 | 0.023 | 1 | 3250 | 3070 |

**Table A.5**

Summary of estimated parameters of the transition of thought from TC1 to TC5.

| Parameters | Estimate | Est. error | l-95% CI | u-95% CI | *R̂* | Bulk_ESS | Tail_ESS |
| --- | --- | --- | --- | --- | --- | --- | --- |
| Intercept | -0.442 | 0.346 | -1.175 | 0.195 | 1 | 2962 | 2600 |
| Score | -0.006 | 0.007 | -0.019 | 0.007 | 1 | 3406 | 2692 |
| Vibration | 0.134 | 0.361 | -0.559 | 0.861 | 1 | 3271 | 2608 |
| Sex | 0.204 | 0.234 | -0.260 | 0.650 | 1 | 3913 | 2604 |
| Score:Vibration | -0.001 | 0.008 | -0.016 | 0.014 | 1 | 3203 | 2560 |

**Fig. A.7.**

**Table A.6**

Summary of estimated parameters of the transition of thought from TC2 to TC1.

| Parameters | Estimate | | Est. error | l-95% CI | u-95% CI | *R̂* | Bulk_ESS | Tail_ESS |
| --- | --- | --- | --- | --- | --- | --- | --- | --- |
| Intercept | | -0.245 | 0.358 | -0.988 | 0.426 | 1 | 2216 | 2710 |
| Score | | -0.004 | 0.007 | -0.017 | 0.009 | 1 | 1795 | 2452 |
| Vibration | | -1.123 | 0.412 | -1.931 | -0.360 | 1 | 2586 | 2615 |
| Sex | | 0.242 | 0.301 | -0.363 | 0.834 | 1 | 1954 | 2259 |
| Score:Vibration | | 0.015 | 0.008 | -0.001 | 0.030 | 1 | 2576 | 2788 |

**Table A.7**

Summary of estimated parameters of the transition of thought from TC4 to TC1.

| Parameter | Estimate | Est. error | l-95% CI | u-95% CI | *R̂* | Bulk_ESS | Tail_ESS |
| --- | --- | --- | --- | --- | --- | --- | --- |
| Intercept | -0.680 | 0.420 | -1.570 | 0.090 | 1 | 2504 | 2927 |
| Score | 0.000 | 0.010 | -0.020 | 0.010 | 1 | 3062 | 2661 |
| Vibration | -0.800 | 0.540 | -1.900 | 0.210 | 1 | 2915 | 2345 |
| Sex | -0.020 | 0.340 | -0.710 | 0.640 | 1 | 3928 | 2813 |
| Score:Vibration | 0.000 | 0.010 | -0.020 | 0.020 | 1 | 2846 | 2423 |

**Table A.8**

Summary of estimated parameters of the transition of thought from TC5 to TC1

| Parameters | Estimate | Est. error | l-95% CI | u-95% CI | *R̂* | Bulk_ESS | Tail_ESS |
| --- | --- | --- | --- | --- | --- | --- | --- |
| Intercept | -0.970 | 0.470 | -1.920 | -0.060 | 1 | 2747 | 2796 |
| Score | -0.010 | 0.010 | -0.030 | 0.010 | 1 | 2780 | 3158 |
| Vibration | 0.440 | 0.440 | -0.440 | 1.310 | 1 | 2983 | 2962 |
| Sex | 0.470 | 0.360 | -0.240 | 1.160 | 1 | 2732 | 3162 |
| Score:Vibration | 0.000 | 0.010 | -0.020 | 0.020 | 1 | 2942 | 2896 |

**Fig. A.8.**

**Table A.9**

Summary of estimated parameters of the continuation of TC1.

| Parameters | Estimate | | Est. error | l-95% CI | u-95% CI | *R̂* | Bulk_ESS | Tail_ESS |
| --- | --- | --- | --- | --- | --- | --- | --- | --- |
| Intercept | -0.539 | 0.405 | | -1.389 | 0.223 | 1 | 1171 | 1828 |
| Score | 0.006 | 0.007 | | -0.008 | 0.021 | 1 | 1013 | 1680 |
| Vibration | -0.245 | 0.294 | | -0.826 | 0.333 | 1 | 2442 | 2566 |
| Sex | 0.642 | 0.332 | | -0.015 | 1.311 | 1 | 893 | 1414 |
| Score:Vibration | 0.001 | 0.005 | | -0.009 | 0.012 | 1 | 2386 | 2582 |

**Table A.10**

Summary of estimated parameters of the continuation of TC2.

| Parameters | Estimate | Est. error | l-95% CI | u-95% CI | *R̂* | Bulk_ESS | Tail_ESS |
| --- | --- | --- | --- | --- | --- | --- | --- |
| Intercept | 0.045 | 0.334 | -0.619 | 0.681 | 1 | 1361 | 1624 |
| Score | 0.002 | 0.006 | -0.010 | 0.014 | 1 | 1305 | 1598 |
| Vibration | -0.074 | 0.248 | -0.560 | 0.401 | 1 | 3223 | 2866 |
| Sex | 0.526 | 0.283 | -0.008 | 1.090 | 1 | 1188 | 1767 |
| Score:Vibration | -0.005 | 0.005 | -0.014 | 0.005 | 1 | 3337 | 2958 |

**Table A.11**

Summary of estimated parameters of the continuation of TC4.

| Parameters | Estimate | Est. error | l-95% CI | u-95% CI | *R̂* | Bulk_ESS | Tail_ESS |
| --- | --- | --- | --- | --- | --- | --- | --- |
| Intercept | 1.131 | 0.275 | 0.584 | 1.671 | 1 | 3186 | 2826 |
| Score | -0.006 | 0.005 | -0.016 | 0.005 | 1 | 3075 | 2867 |
| Vibration | -0.700 | 0.356 | -1.384 | 0.007 | 1 | 2654 | 2981 |
| Sex | -0.234 | 0.199 | -0.619 | 0.153 | 1 | 4158 | 3043 |
| Score:Vibration | 0.011 | 0.007 | -0.003 | 0.025 | 1 | 2772 | 2840 |

**Fig. A.9.**

**Table A.12**

Summary of estimated parameters of highly consistent 5.

| Parameters | Estimate | Est. error | l-95% CI | u-95% CI | *R̂* | Bulk_ESS | Tail_ESS |
| --- | --- | --- | --- | --- | --- | --- | --- |
| Intercept | -0.614 | 0.350 | -1.310 | 0.070 | 1 | 2224 | 2481 |
| Score | 0.006 | 0.006 | -0.006 | 0.017 | 1 | 2307 | 2269 |
| Vibration | 0.314 | 0.296 | -0.271 | 0.888 | 1 | 3536 | 3265 |
| Sex | 0.445 | 0.264 | -0.054 | 0.970 | 1 | 2168 | 2966 |
| Score:Vibration | 0.001 | 0.005 | -0.009 | 0.012 | 1 | 3308 | 3193 |

**Fig. A.10.**

**Table A.13**

Summary of estimated parameters of HF.

| Parameters | Estimate | Est. error | l-95% CI | u-95% CI | *R̂* | Bulk_ESS | Tail_ESS |
| --- | --- | --- | --- | --- | --- | --- | --- |
| Intercept | 1898.438 | 200.654 | 1511.544 | 2304.584 | 1 | 1074 | 1722 |
| Vibration | 4.242 | 31.018 | -33.572 | 80.028 | 1 | 882 | 306 |
| Sex | 1.926 | 23.976 | -23.113 | 46.434 | 1 | 784 | 295 |

**Table A.14**

Summary of estimated parameters of LF/HF.

| Parameters | Estimate | Est. error | l-95% CI | u-95% CI | *R̂* | Bulk_ESS | Tail_ESS |
| --- | --- | --- | --- | --- | --- | --- | --- |
| Intercept | 1.229 | 0.139 | 0.954 | 1.504 | 1 | 2809 | 2799 |
| Vibration | -0.116 | 0.112 | -0.332 | 0.103 | 1 | 8156 | 3238 |
| Sex | 0.264 | 0.237 | -0.212 | 0.727 | 1 | 2533 | 2822 |

**Fig. A.11.**

**Instruction of the thought probes of the VT.**

During the experiment, prompts will appear asking about the content of your immediate thoughts. In such cases, please intuitively select one of the following six options by pressing the corresponding number key that best represents the most dominant thought in your mind:

Task focus: This state indicates your concentration on the task; that is, focusing on responding to stimuli, such as pressing keys when presented with lines.

Task-related: This state involves thinking about aspects related to the task, such as the elapsed time during the task or your performance.

Peripheral stimuli: This state refers to thinking about external environmental factors, like sounds heard during the task.

Internal-body states: This state involves thinking about bodily sensations during the experiment, such as body temperature, pain, or hunger.

Task-unrelated: This state denotes thoughts unrelated to the task, such as recalling past events or making plans for the future.

Absent-mindedness: This state indicates a mental state in which you are not particularly focused on anything specific and might be daydreaming.

When you respond to the actual questions, the screen will display the number, the category name of the thought, and typical examples of each category.

Do you have any questions?

Further, you will be asked about contemplation and consistency of the reported thoughts. Contemplation refers to how deeply you were thinking about the thought you answered, while consistency refers to the level of the coherence of the content of your thought.

For contemplation, please select one of the following:

1. Not very much.

2. Deeply.

3.Very deeply.

For consistency, please select one of the following:

1. Thinking about a single matter.

2. Thinking about multiple contents and their semantic relevance was high.

3. Thinking about multiple contents and their semantic relevance was low.

Consistency is a bit of a difficult concept to grasp, so I will explain it with specific examples. For example, if you were thinking about how much longer the task would continue or if you made a mistake in the previous response, you would choose 2. However, if you were thinking about watching a recorded drama before going to bed or waking up early tomorrow for the 1st lecture, you would choose 3.
